# O02 Hospitalized patients’ perspectives on antimicrobial resistance and stewardship: a mixed-methods study across six clinical groups

**DOI:** 10.1093/jacamr/dlag102.002

**Published:** 2026-06-26

**Authors:** Benson Jacob, Anumol Kurian, Aisling Walsh, Eoghan de Barra

**Affiliations:** Royal College of Surgeons in Ireland, Dublin, Ireland; Health Service Executive, Dublin, Ireland; Department of Public Health and Epidemiology, Royal College of Surgeons in Ireland, Dublin, Ireland; Department of International Health and Tropical Medicine, Royal College of Surgeons in Ireland Dublin, Ireland

## Abstract

**Background:**

Antimicrobial stewardship (AMS) is critical to optimizing antibiotic use and combating antimicrobial resistance (AMR). Yet, hospital stewardship is primarily framed around prescribers and clinical outcomes, with limited attention to patient perspectives. Understanding what matters to patients during prescribing, review, and discontinuation of antimicrobials is essential for patient-centred stewardship. This mixed-method study in a 1000-bed academic hospital explored hospitalized patients’ knowledge, understanding, and expectations of antimicrobial treatment and AMS across six clinical groups: cystic fibrosis, orthopaedics, renal, internal medicine, oncology/haematology, and outpatient parenteral antibiotic therapy (OPAT).

**Methods:**

An explanatory sequential design was employed. Phase 1 involved a 42-item questionnaire administered to 360 patients (*n*=60 per group) to assess AMR/AMS awareness, antimicrobial knowledge and behaviours, comfort in questioning healthcare professionals, and preferred information sources. Descriptive statistics, inferential tests, and logistic regression identified predictors of awareness and knowledge; Phase 2 comprised semi-structured interviews with 28 purposively sampled patients to expand on the quantitative findings. Data were analysed using the Framework Method.

**Objectives:**

To examine hospitalized patients’ understanding, perceptions, and behaviours regarding AMR and AMS, and the factors influencing them. To explore patients’ confidence and willingness to engage with healthcare professionals about antibiotic prescribing and identify what matters most to them in antimicrobial treatment and stewardship.

**Results:**

Quantitative findings showed high AMR awareness (63.6%) but very low AMS awareness (10.6%). Education significantly predicted awareness and higher knowledge scores (β=0.072, t=6.642, *P*<0.001), while clinical group and current antibiotic use were not. Patients generally demonstrated responsible antibiotic behaviours, though advice-seeking preferences and prescribing expectations varied across groups, reflecting differing trust and informational needs. AMR awareness was highest in the renal group, followed by the cystic fibrosis group. Clinical groups demonstrated higher AMR awareness and more positive AMS behaviours, likely facilitated by frequent interactions and consistent support from their clinical teams. Across groups, participants wanted standardized, accessible information on AMR and AMS. Knowledge of diagnostic tests was poor, and patients were generally hesitant to ask clinicians about diagnostic tests performed prior to antibiotic prescribing

**Conclusions:**

Patients recognize antimicrobial resistance as an important issue and are willing to discuss their infection management, yet stewardship-specific knowledge gaps persist. Patients’ engagement with AMS is shaped by knowledge, communication opportunities, and trust in their clinical team. Education emerged as the strongest predictor, while qualitative insights revealed contextual mechanisms underpinning patient perspectives. These findings highlight the importance of patient engagement in decision-making and co-formulated care plans, as well as the value of greater education on AMR and AMS. Hospital antimicrobial stewardship programmes should consider integrating structured, speciality-sensitive, and education-adapted patient engagement strategies to enhance informed and meaningful participation in antibiotic decision-making

